# Effects of immediate loading directionality on the mechanical sensing protein PIEZO1 expression and early-stage healing process of peri-implant bone

**DOI:** 10.1186/s12938-024-01223-1

**Published:** 2024-03-19

**Authors:** Chuanyuan Mao, Weijun Yu, Guanglong Li, Ziyuan Xu, Yuhua Gong, Min Jin, Eryi Lu

**Affiliations:** Department of Stomatology, Renji Hospital, Shanghai Jiao Tong University School of Medicine, 160 Pujian Road, Shanghai, 200127 China

**Keywords:** Peri-implant bone, Immediate loading directionality, PIEZO1, Early stage, Healing process

## Abstract

**Background:**

The reduced treatment time of dental implants with immediate loading protocol is an appealing solution for dentists and patients. However, there remains a significant risk of early peri-implant bone response following the placement of immediately loaded implants, and limited information is available regarding loading directions and the associated in vivo characteristics of peri-implant bone during the early stages. This study aimed to investigate the effects of immediate loading directionality on the expression of mechanical sensing protein PIEZO1 and the healing process of peri-implant bone in the early stage.

**Methods:**

Thirty-two implants were inserted into the goat iliac crest models with 10 N static lateral immediate loading applied, followed by histological, histomorphological, immunohistochemical, X-ray microscopy and energy dispersive X-ray spectroscopy evaluations conducted after 10 days.

**Results:**

From evaluations at the cellular, tissue, and organ levels, it was observed that the expression of mechanical sensing protein PIEZO1 in peri-implant bone was significantly higher in the compressive side compared to the tensile side. This finding coincided with trends observed in interfacial bone extracellular matrix (ECM) contact percentage, bone mass, and new bone formation.

**Conclusions:**

This study provides a novel insight into the immediate loading directionality as a potential influence factor for dental implant treatments by demonstrating differential effects on the mechanical sensing protein PIEZO1 expression and related early-stage healing processes of peri-implant bone. Immediate loading directions serve as potential therapeutic influence factors for peri-implant bone during its early healing stage.

**Supplementary Information:**

The online version contains supplementary material available at 10.1186/s12938-024-01223-1.

## Background

Dental implants have been demonstrated as a reliable treatment alternative for replacing missing teeth, and the current trend is toward to reduce implant loading times [1]. Despite studies on the immediate loading implantation have been carried out with high success rates, there remains a significant risk of early peri-implant bone response in immediately loaded implants [1, 2]. Except for the paucity of available implant models under different directions of immediate loading, there still exists the limitation that the current studies lack comprehensively revealing the healing process and mechanical sensing protein information within peri-implant bone during the early stage.

The initial peri-implant bone formation can be observed on the surface of pristine bone as early as 1 week after implantation, which plays a crucial role in early-stage osseointegration and is strongly influenced by mechanical loading factors [3–5]. The mineralized extracellular matrix (ECM), where most bone cells are embedded, serves as the mechanical microenvironment for bone tissue and presents a combination of various biophysical stimuli [6]. Mechanical cues from the ECM can be transduced into biochemical signals to modulate both cells and their microenvironment, thereby regulating tissue progression. Moreover, these processes rely on mechanosensitive cation channels [7].

Recently, the evolutionarily conserved PIEZO cation channel family (PIEZO1 and PIEZO2 proteins) has been identified as crucial mediator in multiple aspects of mammalian mechanotransduction [8]. Expressed by bone tissue, PIEZO1 plays a critical role in sensing various types of mechanical stresses, including static pressure, membrane stretch and shear stress [9]. It further regulates bone remodeling by converting mechanical stimuli into biochemical signals to modulate the migration, proliferation, and differentiation of osteoblasts. Additionally, it is emerging as a potential novel therapeutic mechanosensor for bone healing process [9, 10]. However, it remains unknown whether PIEZO1 expression occurs in peri-implant bone tissue under different implant loading directions during the early healing stage. We hypothesize that gaining a better understanding of the mechanical sensing protein PIEZO1 expression and its related healing state occurring at peri-implant bone will ultimately lead to improved design strategies for clinical implant therapy based on mechanobiology principles.

Based on the Wolff’s Law, which states that “bone remodels according to the forces, stress or load, applied upon it”, relevant definitive changes of bone can be observed at inner cellular, tissue, and external organ levels when osseous functions are modified [11]. Furthermore, the effects of loading on bone may vary depending on its biological state; thus peri-implant bone could be sensitive to loading during both healing and damaged states [12]. The masticatory force consists of various directional components, and when dealing with cases of insufficient bone, tilted implants may be designed and subjected to non-axial loading [13]. Therefore, an immediately lateral loaded implant model was established in goat iliac crest mimicking human alveolar bone conditions [14, 15], aiming to investigate the effects of immediate loading directionality on peri-implant bone. Based on the action directionality of loading transduction, the regions surrounding implants were divided into two major sides: compressive side and tensile side [16]. Systematic evaluations were conducted at 10 days post-implantation using histology, histomorphology, immunohistochemistry, X-ray microscopy and energy dispersive X-ray spectroscopy (EDX) analysis to assess mechanical sensing protein PIEZO1 expression and related healing process of peri-implant bone. This study provides a novel insight into the immediate loading directionality as a potential influence factor in managing mechanical sensing protein PIEZO1 expression and early healing process of peri-implant bone in immediately loaded implant therapy.

## Results

### Implant surface characterization and clinical observations of experimental animals

**Fig. 1:**
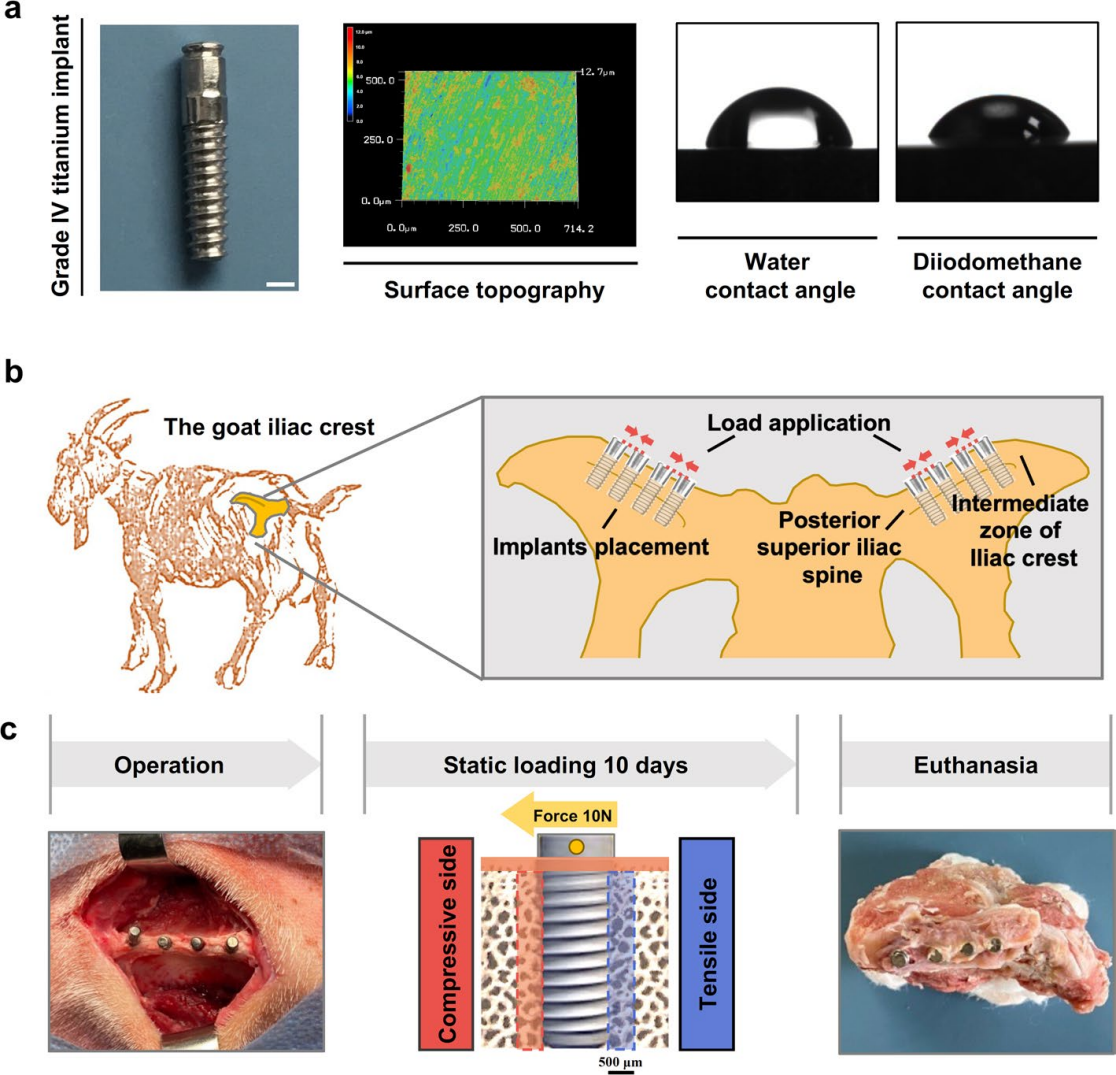
Study design and timeline. **a** Design of the Grade IV titanium implant and its surface characterization. Scale bar, 2 mm. **b** Illustration of the iliac crest and the static load on implants in the goat model. **c** Operation: implants insert parallel to each other with platform at iliac crest level. Static loading method: orthodontic Ni–Ti spring apply continuous static lateral force to the implants. Euthanasia and sample collection

As illustrated in Fig. 1, the study design and timeline were presented. The implant surface topography is shown in Fig. 1a, with an average roughness (Ra) of 0.774 μm (Table 1). The hydrophilicity of implant material was assessed by measuring water and diiodomethane contact angels, which were found to be 75.4° and 56.6°, respectively (Fig. 1a, Table 1). During the experimental period, all goat remained in good general health without exhibiting any symptoms of wound complications or discomfort. After 10 days of surgery, no signs of adverse tissue reactions or inflammation were observed around the surgical sites, and all implant specimens were retrieved (Fig. 1b, c).

**Table 1:** The surface characterization of implant (*n* = 4. Data are mean ± SD)

Water contact angle (°)	Diiodomethane contact angle (°)	Surface roughness Ra (μm)
75.4 ± 3.4	56.6 ± 6.2	0.774 ± 0.030

### The comparison of bone ECM and PIEZO1 expression between the compressive and tensile sides of peri-implant bone

**Fig. 2:**
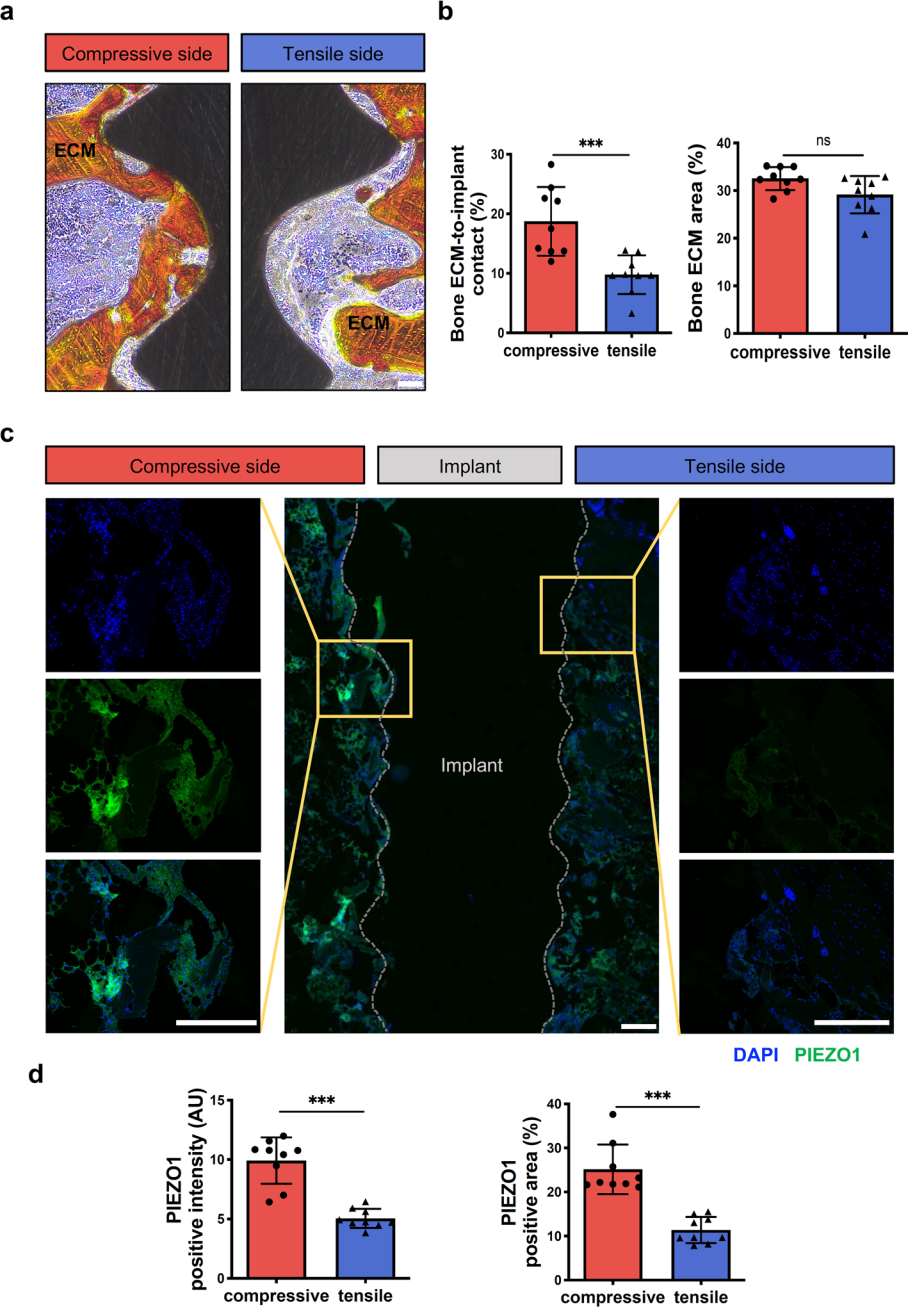
The comparison of peri-implant bone ECM and PIEZO1 expression between the compressive and tensile sides of peri-implant bone. **a** Peri-implant bone ECM was observed by VG staining in the compressive and tensile sides. Bone ECM (orange), the implant (black). Scale bar, 100 μm. **b** The bone ECM-to-implant contact (%) and the peri-implant bone ECM area (%) were quantified at ROIs in the compressive and tensile sides (*n* = 9, Mann–Whitney test). **c** Immunofluorescence staining of PIEZO1 in the compressive and tensile sides. PIEZO1 (green), DAPI (blue). Scale bar, 500 μm. **d** Qualification of intensity and area fraction of positive PIEZO1 cells at ROIs in the compressive and tensile sides (*n* = 9, Mann–Whitney test). All data are represented as mean ± SD. ***p < 0.001. *NS* no significance

The cell–ECM connections play a crucial role in the mechanotransduction mechanism from extracellular mechanical stimuli to nuclear responses [17]. According to the literature, organic collagen and inorganic hydroxyapatite are the primary components of bone, with collagen being the main component of bone ECM [18]. To investigate whether the percentage of bone ECM-to-implant contact and the area of bone ECM were different between the compressive and tensile sides of peri-implant bone, Van Gieson's picrofuchsin (VG) staining was used for collagen analysis [19]. As shown in Fig. 2a, significantly more bone ECM contact with implants was observed in the compressive side compared to the tensile side. Consistently, there was a higher percentage of bone ECM-to-implant contact in the compressive side than in the tensile side (Fig. 2b). Additionally, no significant difference was found in peri-implant bone ECM area between both sides (Fig. 2b).

Since the mechanical sensing protein PIEZO1 acts as a mechanosensor in bone tissue and exhibits positive correlation with loading transduction state [9], further immunofluorescent analysis was conducted to investigate the PIEZO1 expression of peri-implant bone in both compressive and tensile sides. As shown in Fig. 2c, d, the PIEZO1 expression (including the intensity and area fraction) was significantly upregulated in the compressive side compared to the tensile side of peri-implant bone. Collectively, these results indicated that the PIEZO1 expression coincided with the tendency of bone ECM-to-implant contact percentage and suggested that immediate loading directionality may have distinct effects on the early-stage healing process of peri-implant bone.

### The comparison of bone reconstructed images and bone volumetric parameters between the compressive and tensile sides of peri-implant bone

**Fig. 3:**
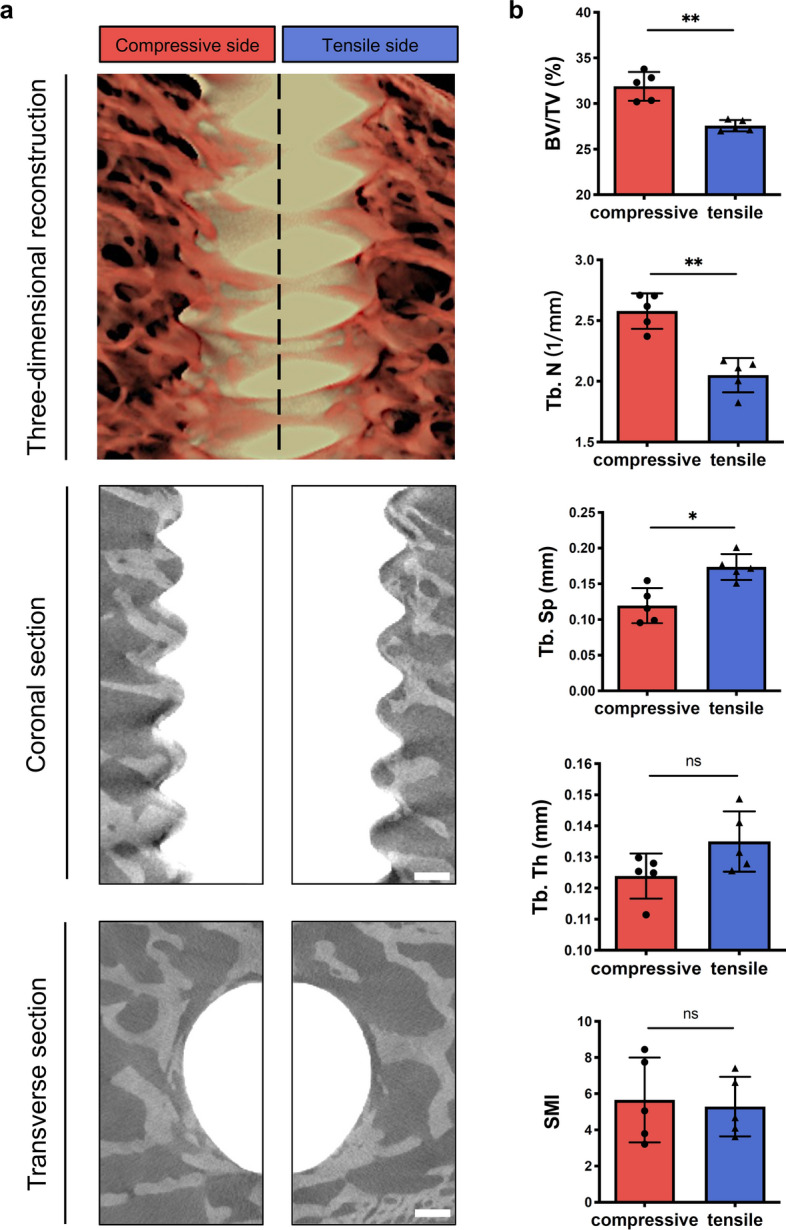
The comparison of bone reconstructed images and bone volumetric parameters between the compressive and tensile sides of peri-implant bone. **a** Representative three-dimensional reconstructed images, coronal section and transverse section images in the compressive and tensile sides. Scale bar, 500 μm. **b** Analysis of X-ray microscopy volumetric parameters BV/TV, Tb.N, Tb.Sp, Tb.Th and SMI at ROIs in the compressive and tensile sides (*n* = 5, Mann–Whitney test). All data are represented as mean ± SD. *p < 0.05. **p < 0.01. *NS* no significance

To further investigate the precise microstructure characteristics of peri-implant bone in different loading direction sides, X-ray microscopy and bone morphometry indices were performed. The compressive side exhibited higher peri-implant bone mass compared to the tensile side (Fig. 3a). Additionally, the compressive side showed significantly higher values for bone volumetric parameters bone volume/total volume (BV/TV) and trabecular number (Tb.N) of peri-implant bone than the tensile side, and trabecular separation (Tb.Sp) of peri-implant bone was lower in the compressive side compared to the tensile side. However, no significant differences were found in trabecular thickness (Tb.Th) and structure model index (SMI) among the both sides (Fig. 3b). Thus, these results indicated that there are differences in the microstructure between two sides of peri-implant bone, with increased peri-implant bone mass observed in the compressive side compared to the tensile side.

### The comparison of healing state between the compressive and tensile sides of peri-implant bone

**Fig. 4:**
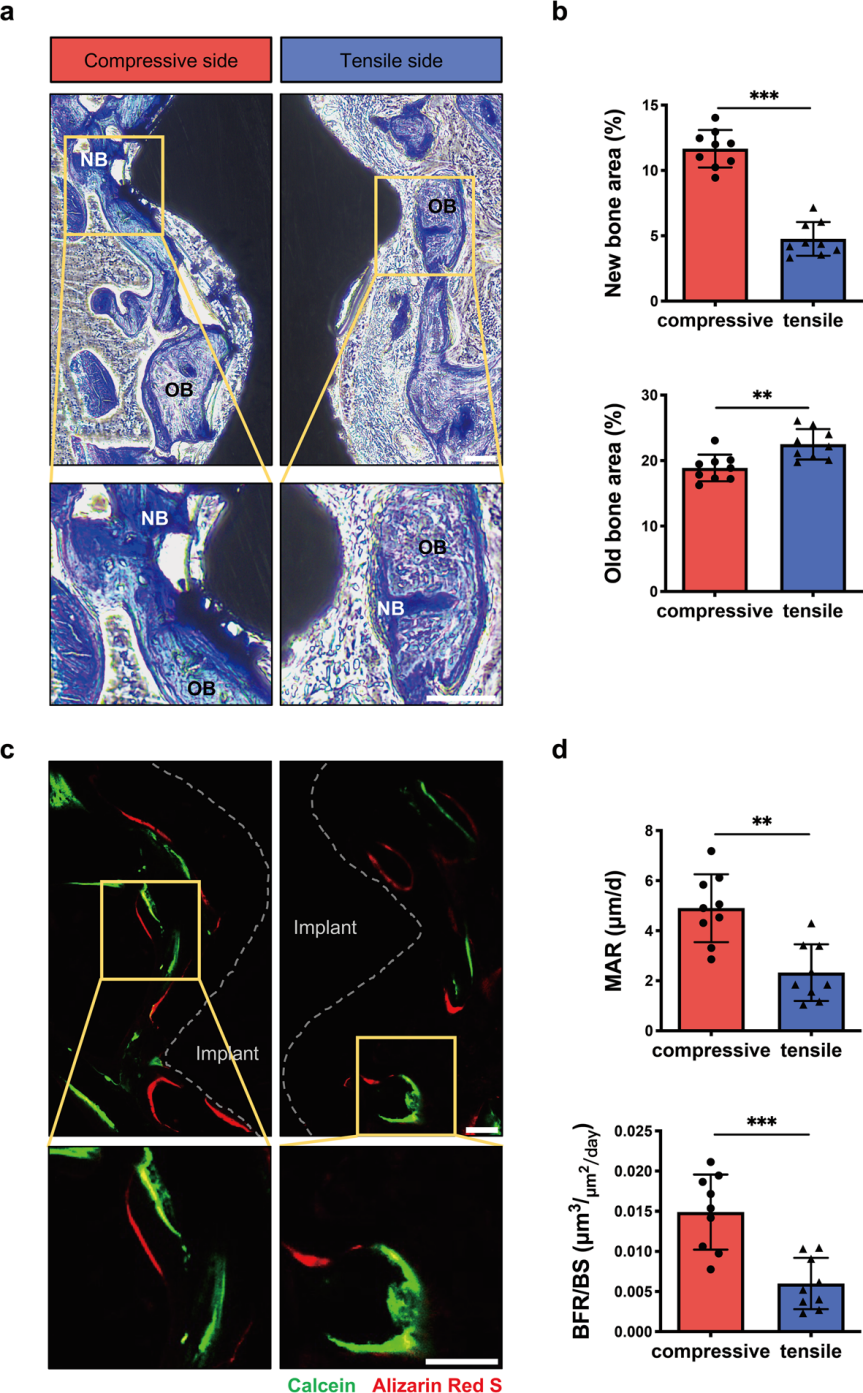
The comparison of bone healing state between the compressive and tensile sides of peri-implant bone. **a** Toluidine blue histological staining of the peri-bone in the compressive and tensile sides. OB: old bone (light blue), NB: new bone (dark blue), and the implant (black). Scale bar, 100 μm. **b** Qualification of new bone and old bone area (%) at ROIs in the compressive and tensile sides (*n* = 9, Mann–Whitney test). **c** Sequential fluorescent labeled images of the peri-bone in the compressive and tensile sides, showing calcein (green) and alizarin red (red). Scale bar, 100 μm. **d** Analysis of the dynamic parameter MAR and BFR/BS at ROIs in the compressive and tensile sides (*n* = 9, Mann–Whitney test). All data are represented as mean ± SD. **p < 0.01. ***p < 0.001

As the PIEZO1 expression and the microstructure of peri-implant bone were obviously different between the two sides, we hypothesized that the immediate loading directionality would also influence the healing state of peri-implant bone during early stage. To test this hypothesis, toluidine blue staining and sequential fluorescent labeling assay were performed. As shown in Fig. 4a, a larger area of new bone formation was observed in the compressive side compared to the tensile side. Furthermore, the quantitative analysis confirmed these observations. In contrast, there was less old bone area in the compressive side than tin he tensile side (Fig. 4b). Similarly, the dynamic parameter mineral apposition rate (MAR) and bone formation rate/bone surface (BFR/BS) of peri-implant bone in the compressive side were higher than that in the tensile side (Fig. 4c, d). In addition, no obvious tartrate-resistant acid phosphatase staining (TRAP) positive osteoclasts were observed in peri-implant bone of both sides during this stage (Additional file 1: Fig. S1). These data suggested that more new bone formation was found in the compressive side relative to the tensile side.

**Fig. 5:**
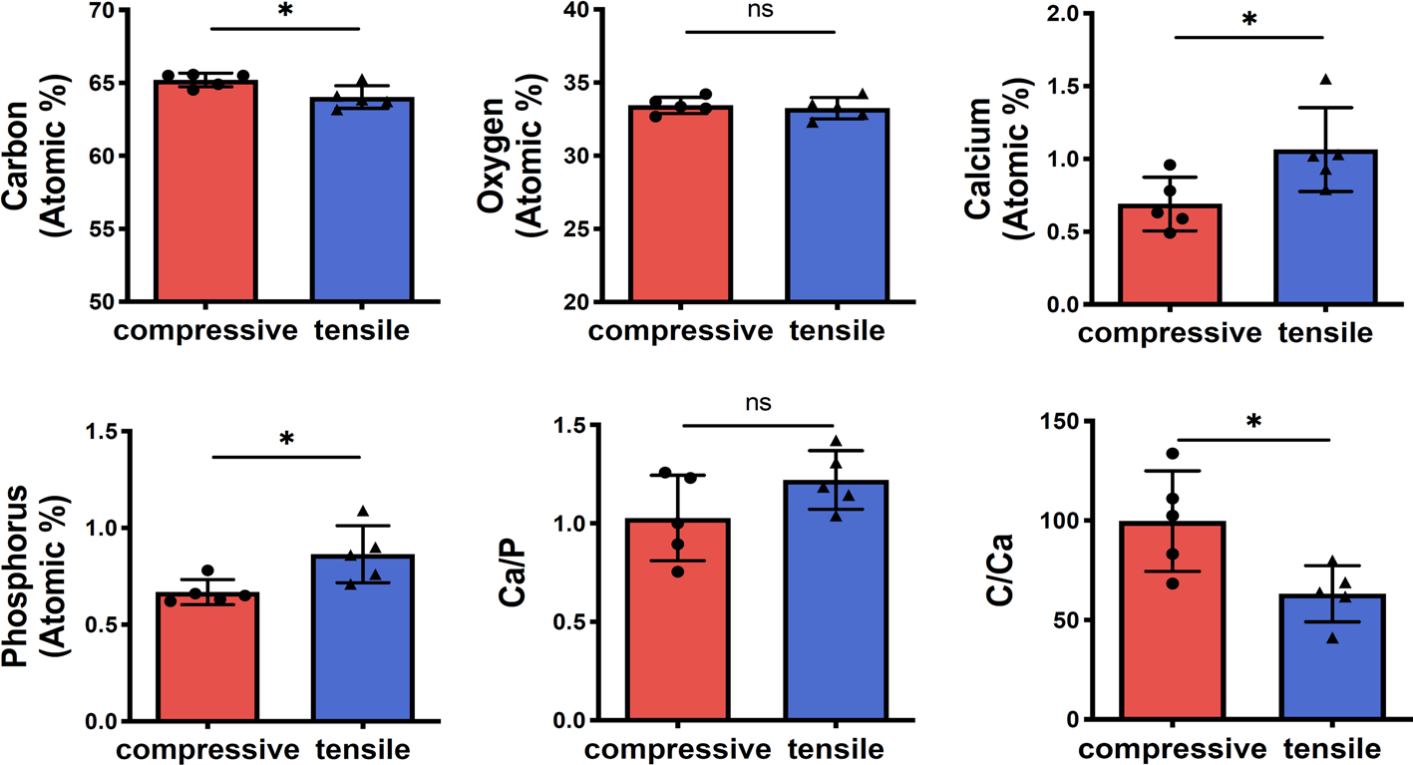
The comparison of elements between the compressive and tensile sides of peri-implant bone. Elemental analysis by EDX showing the carbon, oxygen, calcium, and phosphorus levels, Ca/P and C/Ca ratios at ROIs in the compressive and tensile sides (*n* = 5, Mann–Whitney test). All data are represented as mean ± SD. *p < 0.05. *NS* no significance

To further elucidate the composition of the dominant elements in peri-implant bone, the EDX was used to analyze the elemental content of calcium, phosphorus, oxygen and carbon. The calcium and phosphorus content of peri-implant bone were lower in the compressive side than that in the tensile side; conversely, carbon content was higher (Fig. 5). What’s more, the carbon/calcium (C/Ca) ratio of peri-implant bone was higher in the compressive side compared to the tensile side, and no significant difference was found in the calcium/phosphorus (Ca/P) ratio between both sides (Fig. 5). Taken together, these results indicated that the peri-implant bone was less mature in the compressive side compared to the tensile side, which was consistent with the observation of toluidine blue staining.

## Discussion

The contemporary treatment and fabrication concepts aim to minimize therapy durations and medical visits while maintaining optimum clinical and patient-related outcomes of therapy [20]. Immediate loading therapeutic schedule offer an attractive solution for both clinicians and patients by reducing overall implant treatment time [21]. Immediate loading refers to placing the implant into function within the first week after its placement [22]. However, immediately loaded implant was associated with a higher incidence of implant failure compared to conventional delay loaded implant [23]. Occlusal contact on the implant superstructures is a crucial factor for successful immediately loaded implant therapy [24]. Moreover, nature masticatory forces can load on implants in different directions [13]. Besides, few in vivo studies have been published documenting the effects of immediate loading directionality on peri-implant bone characteristics, especially limited information focus on cellular, tissue, and external organ analyses during the early stages. Herein, we demonstrate that immediate loading directionality is a therapeutic influence factor affecting the mechanical sensing protein PIEZO1 expression at cellular level and impacting early-stage healing processes of peri-implant bone at tissue and organ levels. In the present research, distinct differences in peri-implant bone healing state were observed between compressive and tensile sides which coincided with the tendency of the interfacial bone ECM contact percentage and the PIEZO1 expression in peri-implant bone.

Several major factors influence the healing process of peri-implant bone, including local stimuli and mechanical stress, bone cell activity, and the balance between anabolic and catabolic local factors affecting bone formation and remodeling [25]. The early formation of peri-implant trabecular bone starts at 10–14 days after implantation, which ensures tissue mechanical anchorage to the implant. Direct mechanical anchorage of implant is still lacking in the early stage of implantation [25]. Although there was a higher percentage of bone ECM-to-implant contact in the compressive side compared to the tensile side during this early stage (Fig. 2a, b), both were at low levels compared to long term healing periods [26, 27]. Therefore, it appears that lack of direct mechanical anchorage may affect load transduction to peri-implant bone. Furthermore, the present study demonstrated that loading directionality can influence the mechanical sensing protein PIEZO1 expression in peri-implant bone during this early stage, and the PIEZO1 expression was significantly upregulated in the compressive side compared to the tensile side (Fig. 2c, d). As previously demonstrated in a model study on ECM-mediated sensitization of PIEZO1 receptors, it was observed that mechanical force pulling the cellular membrane without the presence of ECM proteins did not effectively activate PIEZO1 receptors. However, a certain degree of mechanical force pushing the membrane could activate these receptors [28]. Additionally, osteoblasts exhibits a preference for attaching to protein-functionalized areas [29]. Therefore, these findings suggest that the immediate loading directionality plays an important role in influencing early-stage healing processes of peri-implant bone when the direct mechanical anchorage of implant is lacking.

Mechanotransduction is a key process involved in various developmental, physiological, and pathological processes within bone tissue [30]. Among mechanosensitive cation channels, PIEZO1 serves as one of the important participants in mechanotransduction and its expression level is influenced by mechanical loading [30, 31]. A recent study reveals that PIEZO1 plays a crucial role in converting mechanical stimuli into osteogenesis of bone cells and is essential for bone formation [31]. In this study, the expression levels of PIEZO1 in both compressive and tensile sides of peri-implant bone were associated with bone healing manner observed through bone histomorphology and morphology analysis (Figs. 3, 4).

Bone is highly dynamic connective tissue, and its healing processes are tightly regulated by mechanical stimuli associated with both renewal of old or damaged bone and the formation of new bone [32]. A larger area of new bone formation was observed in the compressive side compared to the tensile side (Fig. 4a, d). Furthermore, the percentage of bone-to-implant contact is influenced by the speed of new bone apposition onto the implant surface [33]. In addition, EDX elemental analysis revealed that in the compressive side, peri-implant bone had higher carbon content and C/Ca ratio compared to the tensile side, while calcium and phosphorus content showed an inverse trend (Fig. 5). The variation in elemental content of the main bone partially indicates differences in tissue maturity, whereas the C/Ca ratio presents the carbon content of organic matrix and the carbonate content of bone apatite, which may increase during the early mineralization stages [34].

The present study has some limitations. Considering the potential detrimental effects of excessive and cyclic load conditions on peri-implant bone [35, 36], a mild static load of 10 N was chosen in this study. Since newly formed bone contact with the implant surface can be observed at one week after implantation [5], the observation period was set at an early stage of 10 days. Future studies should place emphasis on sequential factors to further elucidate the mechanism involved in immediately loaded implant therapy.

## Conclusions

The present study has demonstrated that the immediate loading directionality exerts differential effects on the mechanical sensing protein PIEZO1 expression and related healing process of peri-implant bone during the early implantation stage. From cellular, tissue to organ level evaluations, the expression of mechanical sensing protein PIEZO1 in peri-implant bone was significantly higher in the compressive side compared to the tensile side, which coincided with the tendency of the interfacial bone ECM contact percentage, bone mass and new bone formation. This study provides a novel insight into the immediate loading directionality as a therapeutic influence factor. In addition, clinicians should pay attention to loading directions when adjusting occlusion during early implantation stages for immediately loaded implants.

## Methods

### Animals and study design

According to the literature, the goat crest exhibits highly similar physiological, pathological and bone structural conditions compared to human alveolar ridge. Additionally, the iliac crest implantation model is reliable for studying biological responses in peri-implant bone while avoiding oral microbiota and masticatory force influences [37, 38]. Four healthy female goats aged between 26 and 30 months with an average weight of 50 kg were used in this study. All the procedures of animal experiments were approved by the Institutional Animal Care and Use Committee of the Ninth People’s Hospital, Shanghai Jiao Tong University School of Medicine (SH9H-2021-A92-1) and conducted in compliance with ARRIVE guidelines [39].

### Implantation procedure, loading method and sample collection

Thirty-two implants were fabricated using commercially pure grade IV titanium by subtractive manufacturing [33]. The implant shape was designed to mimic a standard regular neck implant (Straumann^®^, Switzerland), with all implants measuring 10.0 mm in length and 3.3 mm in diameter. The surface of the implants underwent progressively ground using silicon carbide papers up to 1000 grits. Then the surface topography and roughness of the implant material samples were measured by 3D laser scanning confocal microscope (VK-X 110, Japan), and the surface hydrophobicity was detected using contact angle goniometer (SL200B, USA).

The implant surgery procedures followed detailed descriptions by Schouten et al. [14]. All surgical procedures were performed under sterile conditions with isoflurane general anesthesia. In brief, a lateral incision was made on both sides of the vertebral column just above the anterior superior part of the iliac crest. Then, implant holes were gradually performed using low rotational drills with increasing diameter and under continuous external saline cooling. The distance between each two holes was maintained at 5-mm intervals. Next, four implants were inserted on each side of iliac crest with insertion torque value over 30 Ncm. After implant placement, implants were subjected to lateral 10 N static loading application by connecting each pair with an orthodontic Ni–Ti spring (Ormco, Italy) through grooves made on their abutments. Finally, the closure of incision was performed with 4-0 vicryl suture (Jinhuan, China). To minimize the risks of peri-operative infection and pain, pre- and post-surgical antibiotics as well as post-operative analgesia were administered to all goats involved in this study. 10 days after surgery completion, euthanasia was performed on all goats involved in this study. And the block specimens containing the implant and peri-implant hard tissue were fixed in 4% paraformaldehyde solution.

### Analysis of X-ray microscopy images and bone morphometry indices

To evaluate microstructure of peri-implant bone more precisely, samples were randomly selected for scanning using X-ray microscopy (ZEISS Xradia Versa 520, USA) with the following parameters: an X-ray source voltage of 80 kV, power of 7 W, and pixel size of 10 μm. Then the three-dimensional images of implants and peri-implant bone were reconstructed by XMReconstructor (Carl Zeiss, USA). To evaluate the inner zone of peri-implant bone response, the region of interest (ROI) of peri-implant bone in each tomography was selected from middle 30 layers of implant body and was defined as a rectangle extending 500 μm from the implant surface, with the entire length of the implant screw [40]. Next, the bone morphometry indices were measured from three-dimensional images at ROIs in the compressive and tensile sides, including BV/TV, Tb.N, Tb.Sp, Tb.Th and SMI, which were calculated using CTAn software (Skyscan, Belgium) [41].

### Histological, histomorphological and spectroscopic analysis

The specimens were dehydrated, embedded in methyl methacrylate (Sigma-Aldrich, USA), sliced and ground to 10-μm-thick sections. The ROIs of peri-implant bone in each analysis was defined as a rectangle extending 500 μm from the implant surface, with the entire length of the implant screw. For bone ECM analysis, the sections were stained with VG (Sigma-Aldrich, USA) and visualized via the inverted microscope (Nikon eclipse, Japan). The percentage of bone ECM-to-implant contact and the area of peri-implant bone ECM were quantified using ImageJ software. For bone healing analysis, the sections were stained in toluidine blue, and the histological images were taken under the inverted microscope (Nikon eclipse. Japan). The quantification of new bone and old bone area were obtained using ImageJ software. To record the peri-implant bone healing process, the sequential fluorescent labeling method was set at day 1 and day 8 post-implantation. And 15 mg/kg calcein solution and 20 mg/kg alizarin red solution were administered to goat for mineralized tissue label, respectively. The MAR and BFR/BS were calculated using ImageJ software. To investigate the composition of peri-implant bone, EDX (Hitachi S4800, Japan) was used.

For immunofluorescent and TRAP staining, the specimens were decalcified with 15% PH 7.8 ethylene diamine tetraacetic acid (EDTA), embedded in paraffin and sectioned at 6 μm. Then, the sections were blocked with blocking buffer for 1 h and incubated overnight with PIEZO1 antibody (NBP1-78446, NOVUS). Next, the sections were incubated with the secondary antibody (Alexa Fluor 488, Cell Signal Technology) for 1 h. And 6-diamidino-2-phenylindole (DAPI, Sigma-Aldrich, USA) was used for counterstaining. The intensity and area fraction of positive staining were quantified using ImageJ software. TRAP staining of osteoclasts was performed with a TRAP staining kit (Sigma-Aldrich, USA).

### Statistical analysis

All the experiments were performed at least in triplicate and the data were presented as mean ± SD. Statistical analysis was performed using version 9.0 GraphPad Prism (San Diego, USA). Mann–Whitney test was used for comparison of two independent groups. Significant difference between groups was denoted as: *p < 0.05, ** *p* < 0.01, and ***p < 0.001.

### Supplementary Information

**Additional file 1 Figure S1**. The comparison of osteoclast activity between the compressive and tensile sides in peri-implant bone.

## Data Availability

The datasets used and/or analyzed during the current study are available from the corresponding author on reasonable request.

## Abbreviations

ECM: Extracellular matrix
EDX: Energy dispersive X-ray spectroscopy
BV/TV: Bone volume/total volume
Tb.N: Trabecular number
Tb.Sp: Trabecular separation
Tb.Th: Trabecular thickness
SMI: Structure model index
MAR: Mineral apposition rate
BFR/BS: Bone formation rate/bone surface
TRAP: Tartrate-resistant acid phosphatase
Ca/P: Calcium/phosphorus
C/Ca: Carbon/calcium
ROI: The region of interest
EDTA: Ethylene diamine tetraacetic acid
DAPI: 6-Diamidino-2-phenylindole
Ra: Average roughness

## References

[1] Pardal-Pelaez B, Flores-Fraile J, Pardal-Refoyo JL, Montero J (2021). Implant loss and crestal bone loss in immediate versus delayed load in edentulous mandibles: a systematic review and meta-analysis. J Prosthet Dent.

[2] Zhu Y, Zheng X, Zeng G, Xu Y, Qu X, Zhu M, Lu E (2015). Clinical efficacy of early loading versus conventional loading of dental implants. Sci Rep.

[3] Yu T, Gao H, Liu T, Huang Y, Wang C (2020). Effects of immediately static loading on osteointegration and osteogenesis around 3D-printed porous implant: a histological and biomechanical study. Mater Sci Eng C Mater Biol Appl.

[4] Duyck J, Vandamme K (2014). The effect of loading on peri-implant bone: a critical review of the literature. J Oral Rehabil.

[5] Salvi GE, Bosshardt DD, Lang NP, Abrahamsson I, Berglundh T, Lindhe J, Ivanovski S, Donos N (2015). Temporal sequence of hard and soft tissue healing around titanium dental implants. Periodontology 2000.

[6] Qin L, Liu W, Cao H, Xiao G (2020). Molecular mechanosensors in osteocytes. Bone Res.

[7] Zhou W, Liu X, van Wijnbergen JWM, Yuan L, Liu Y, Zhang C, Jia W (2020). Identification of PIEZO1 as a potential prognostic marker in gliomas. Sci Rep.

[8] Zhao Q, Zhou H, Chi S, Wang Y, Wang J, Geng J, Wu K, Liu W, Zhang T, Dong MQ (2018). Structure and mechanogating mechanism of the Piezo1 channel. Nature.

[9] Wang L, You X, Lotinun S, Zhang L, Wu N, Zou W (2020). Mechanical sensing protein PIEZO1 regulates bone homeostasis via osteoblast-osteoclast crosstalk. Nat Commun.

[10] Zhang G, Li X, Wu L, Qin YX (2021). Piezo1 channel activation in response to mechanobiological acoustic radiation force in osteoblastic cells. Bone Res.

[11] Rossetti PH, Bonachela WC, Rossetti LM (2010). Relevant anatomic and biomechanical studies for implant possibilities on the atrophic maxilla: critical appraisal and literature review. J Prosthodont.

[12] Brunski JB (1999). In vivo bone response to biomechanical loading at the bone/dental–implant interface. Adv Dent Res.

[13] Demenko V, Linetskiy I, Nesvit K, Shevchenko A (2011). Ultimate masticatory force as a criterion in implant selection. J Dent Res.

[14] Schouten C, Meijer GJ, van den Beucken JJ, Spauwen PH, Jansen JA (2010). A novel implantation model for evaluation of bone healing response to dental implants: the goat iliac crest. Clin Oral Implant Res.

[15] Mao C, Yu W, Jin M, Wang Y, Shang X, Lin L, Zeng X, Wang L, Lu E (2022). Mechanobiologically optimized Ti-35Nb-2Ta-3Zr improves load transduction and enhances bone remodeling in tilted dental implant therapy. Bioactive Mater.

[16] Matsuzaki T, Ayukawa Y, Matsushita Y, Sakai N, Matsuzaki M, Masuzaki T, Haraguchi T, Ogino Y, Koyano K (2019). Effect of post-osseointegration loading magnitude on the dynamics of peri-implant bone: a finite element analysis and in vivo study. J Prosthodont Res.

[17] Kardas D, Nackenhorst U, Balzani D (2013). Computational model for the cell-mechanical response of the osteocyte cytoskeleton based on self-stabilizing tensegrity structures. Biomech Model Mechanobiol.

[18] Kerschnitzki M, Wagermaier W, Roschger P, Seto J, Shahar R, Duda GN, Mundlos S, Fratzl P (2011). The organization of the osteocyte network mirrors the extracellular matrix orientation in bone. J Struct Biol.

[19] Nuka S, Zhou W, Henry SP, Gendron CM, Schultz JB, Shinomura T, Johnson J, Wang Y, Keene DR, Ramirez-Solis R (2010). Phenotypic characterization of epiphycan-deficient and epiphycan/biglycan double-deficient mice. Osteoarthritis Cartil OARS Osteoarthritis Res Soc.

[20] Aiquel LL, Pitta J, Antonoglou GN, Mischak I, Sailer I, Payer M (2021). Does the timing of implant placement and loading influence biological outcomes of implant-supported multiple-unit fixed dental prosthesis—a systematic review with meta-analyses. Clin Oral Implant Res.

[21] Gallucci GO, Hamilton A, Zhou W, Buser D, Chen S (2018). Implant placement and loading protocols in partially edentulous patients: a systematic review. Clin Oral Implant Res.

[22] Huynh-Ba G, Oates TW, Williams MAH (2018). Immediate loading vs. early/conventional loading of immediately placed implants in partially edentulous patients from the patients' perspective: a systematic review. Clin Oral Implants Res.

[23] Chen J, Cai M, Yang J, Aldhohrah T, Wang Y (2019). Immediate versus early or conventional loading dental implants with fixed prostheses: a systematic review and meta-analysis of randomized controlled clinical trials. J Prosthet Dent.

[24] Okada Y, Sato Y, Kitagawa N, Uchida K, Osawa T, Imamura Y, Terazawa M (2015). Occlusal status of implant superstructures at mandibular first molar immediately after setting. Int J Implant Dent.

[25] Mavrogenis AF, Dimitriou R, Parvizi J, Babis GC (2009). Biology of implant osseointegration. J Musculoskelet Neuronal Interact.

[26] Thoma DS, Lim HC, Paeng KW, Jung UW, Hammerle CHF, Jung RE (2019). Tissue integration of zirconia and titanium implants with and without buccal dehiscence defects—a histologic and radiographic preclinical study. Clin Oral Implant Res.

[27] Manzano G, Herrero LR, Montero J (2014). Comparison of clinical performance of zirconia implants and titanium implants in animal models: a systematic review. Int J Oral Maxillofac Implants.

[28] Gaub BM, Müller DJ (2017). Mechanical stimulation of piezo1 receptors depends on extracellular matrix proteins and directionality of force. Nano Lett.

[29] Pan CJ, Qin H, Nie YD, Ding HY (2013). Control of osteoblast cells adhesion and spreading by microcontact printing of extracellular matrix protein patterns. Colloids Surf B.

[30] Pei F, Liu J, Zhang L, Pan X, Huang W, Cen X, Huang S, Jin Y, Zhao Z (2021). The functions of mechanosensitive ion channels in tooth and bone tissues. Cell Signal.

[31] Sun W, Chi S, Li Y, Ling S, Tan Y, Xu Y, Jiang F, Li J, Liu C, Zhong G (2019). The mechanosensitive Piezo1 channel is required for bone formation. ELife.

[32] Qin L, He T, Chen S, Yang D, Yi W, Cao H, Xiao G (2021). Roles of mechanosensitive channel Piezo1/2 proteins in skeleton and other tissues. Bone Res.

[33] Bosshardt DD, Chappuis V, Buser D (2017). Osseointegration of titanium, titanium alloy and zirconia dental implants: current knowledge and open questions. Periodontology 2000.

[34] Shah FA, Snis A, Matic A, Thomsen P, Palmquist A (2016). 3D printed Ti6Al4V implant surface promotes bone maturation and retains a higher density of less aged osteocytes at the bone–implant interface. Acta Biomater.

[35] Esaki D, Matsushita Y, Ayukawa Y, Sakai N, Sawae Y, Koyano K (2012). Relationship between magnitude of immediate loading and peri-implant osteogenesis in dogs. Clin Oral Implant Res.

[36] Isidor F (2006). Influence of forces on peri-implant bone. Clin Oral Implant Res.

[37] Schouten C, Meijer GJ, van den Beucken JJ, Leeuwenburgh SC, de Jonge LT, Wolke JG, Spauwen PH, Jansen JA (2010). In vivo bone response and mechanical evaluation of electrosprayed CaP nanoparticle coatings using the iliac crest of goats as an implantation model. Acta Biomater.

[38] Lm W (2015). Animal models for evaluation of bone implants and devices: comparative bone structure and common model uses. Vet Pathol.

[39] Kilkenny C, Browne WJ, Cuthill IC, Emerson M, Altman DG (2010). Improving bioscience research reporting: the ARRIVE guidelines for reporting animal research. PLoS Biol.

[40] Schouten C, Meijer GJ, van den Beucken JJ, Spauwen PH, Jansen JA (2009). The quantitative assessment of peri-implant bone responses using histomorphometry and micro-computed tomography. Biomaterials.

[41] Cao X, Wang C, Yuan D, Chen S, Wang X (2022). The effect of implants loaded with stem cells from human exfoliated deciduous teeth on early osseointegration in a canine model. BMC Oral Health.

