# Supplementary material for: Effects of immediate loading directionality on the mechanical sensing protein PIEZO1 expression and early-stage healing process of peri-implant bone
Source: Biomed Eng Online. 2024 Mar 19;23:36. doi: 10.1186/s12938-024-01223-1 (PMC10953093; doi:10.1186/s12938-024-01223-1)
Supplement: Supplementary file 1 — Additional file 1 Figure S1. The comparison of osteoclast activity between the compressive and tensile sides in peri-implant bone. [file 12938_2024_1223_MOESM1_ESM.pdf]

figure S1

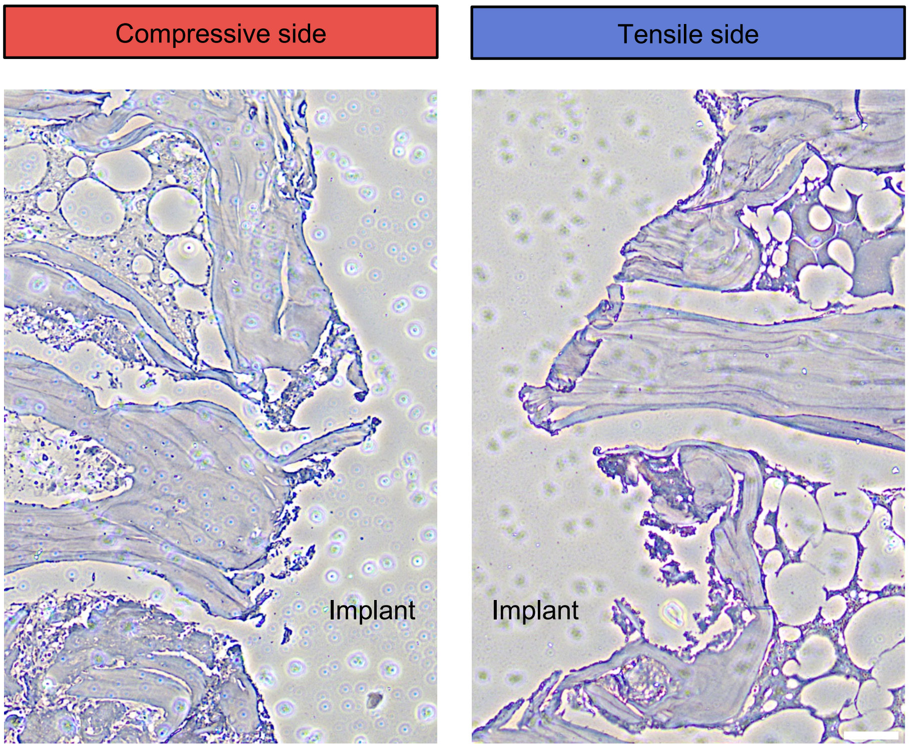

Fig. S1. The comparison of osteoclast activity between the compressive and tensile sides in peri-implant bone. Scale bar, 100 μm.
